# Supplementary material for: Effectiveness and Safety of Ayurvedic Medicines in Type 2 Diabetes Mellitus Management: A Systematic Review and Meta-Analysis
Source: Front Pharmacol. 2022 Jun 8;13:821810. doi: 10.3389/fphar.2022.821810 (PMC9213670; doi:10.3389/fphar.2022.821810)
Supplement: Supplementary file 1 [file DataSheet1.zip › Supplementary Material/Forest and Funnel Plots/Trigonella foenum-graecum/Funnel plots/Funnel plot_Trigonella foenum-graecum_FBG.pdf]

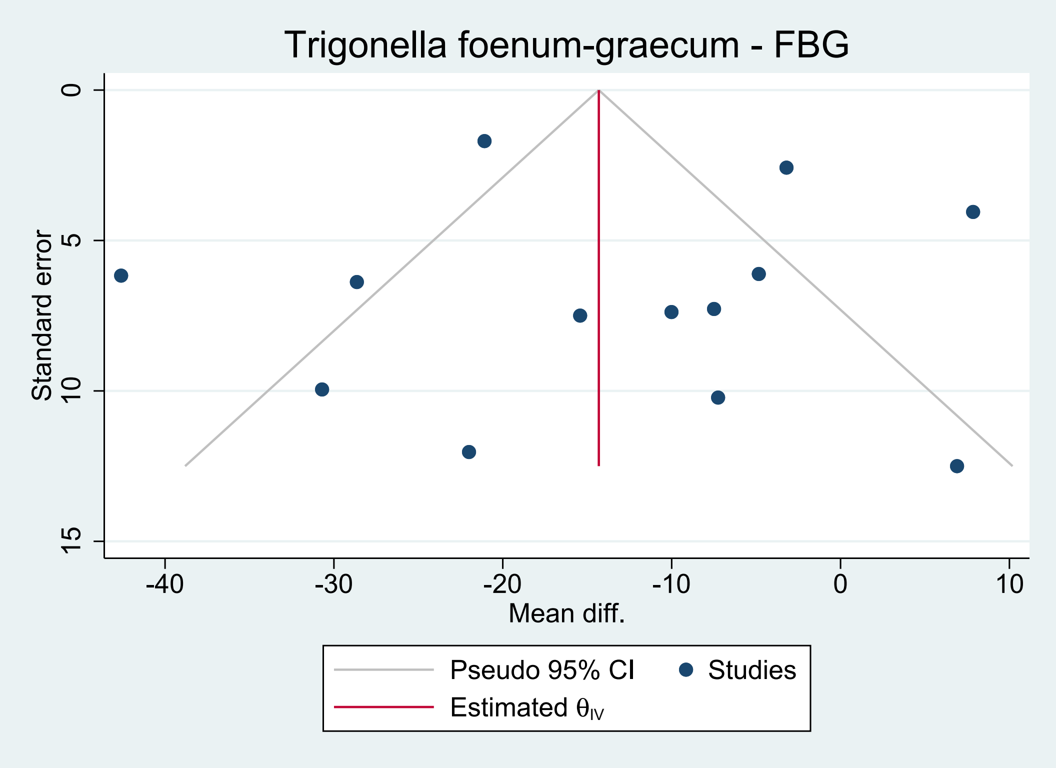

Trigonella foenum-graecum - FBG
Standard error
0
5
10
15
-40
-30
-20
-10
0
10
Mean diff.
Pseudo 95% CI
Studies
Estimated θIV
